# The efficacy and safety of silver needle in the treatment of rheumatoid arthritis: A protocol of randomized controlled trial

**DOI:** 10.1097/MD.0000000000025556

**Published:** 2021-05-07

**Authors:** Jinling Yu, Pingsheng Wang, Cui Nie, Bo Zheng

**Affiliations:** Bayannur City Hospital, Inner Mongolia 015000, China.

**Keywords:** Silver needle therapy, rheumatoid arthritis, Randomized controlled trial, protocol

## Abstract

**Background:**

Rheumatoid arthritis is a kind of chronic crippling disease, the condition is complex, the course of the disease is repeated, seriously affecting the quality of life of patients. Adverse reactions and drug resistance associated with conventional treatment can no longer meet the clinical need. Therefore, complementary and alternative therapies need to be explored. The evidence shows that silver needle therapy has advantages in the treatment of rheumatoid arthritis, but there is a lack of standard clinical studies to verify this conclusion.

**Methods:**

This is a prospective randomized controlled trial to study the efficacy and safety of silver needles in the treatment of rheumatoid arthritis. Approved by the Clinical Research Ethics Committee of our hospital. The patients are randomly divided into a treatment group (silver needle treatment group) or control group (routine western medicine treatment group). The patients are followed up for 2 months after 4 weeks of treatment. Observation indicators include: TCM symptom score, HAQDI score, DAS-28 score, laboratory indicators, adverse reactions and so on. Data will be analyzed using the statistical software package SPSS version 18.0 (Chicago, IL).

**Discussion:**

This study will evaluate the clinical efficacy of a silver needle in the treatment of rheumatoid arthritis. The results of this study will provide a reliable reference for the clinical use of a silver needle in the treatment of rheumatoid arthritis.

**Trial registration:**

OSF Registration number: DOI 10.17605/OSF.IO/4X5QB

## 1 Introduction

Rheumatoid arthritis (RA) is the most common inflammatory arthritis. It is a chronic autoimmune disease characterized by symmetrical, persistent synovitis and destructive polyarthritis.^[1]^ According to 2010 data, the prevalence of RA accounts with 0.24% of the global population.^[2]^ In the United States, the annual loss caused by RA is more than $1 billion.^[3]^ RA is more common in women, and the risk of RA in women is about 3 times higher than that in men. The disease can occur at any age, the peak of incidence is between 30 and 50 years old, and the incidence increases with age, with the highest in women over 65 years old.^[4]^ RA is characterized by symmetrical multi-joint swelling and pain in the hand, wrist, foot and so on. The pathological changes are synovitis and pannus formation of joints, which lead to articular cartilage and bone erosion, joint deformities and loss of function, which is one of the main causes of disability.^[5]^

Rheumatoid arthritis is a disabled chronic disease, the condition is complex, the course of the disease is repeated, there is no specific treatment. At present, the mainstream management schemes for RA include nonsteroidal anti-inflammatory drugs (NSAIDs), conventional synthetic disease modifying anti-rheumatic drugs (csDMARDs), biological disease modifying anti-rheumatic drugs (bDMARDs), target synthetic disease modifying anti-rheumatic drugs (tsDMARDs), glucocorticoids and botanical drugs modified by modification.^[6]^ Most patients can achieve clinical remission after treatment, but there are still some patients who are easy to develop into refractory RA, and almost need to take drugs for life. In addition, the drugs for the treatment of RA not only delay the disease, but also bring great adverse reactions, which are difficult for patients to tolerate both financially and physically.^[7,8]^ Therefore, it is particularly important to seek safe and reliable complementary replacement therapy.

As a traditional Chinese medicine therapy, acupuncture plays an important role in many diseases.^[9]^ Silver needle therapy is similar to traditional acupuncture therapy, but there are some differences. Silver needles are not placed on acupoints, but on muscles, tendons and the fascia, heated by special machines to eliminate aseptic inflammation and relieve pain.^[10]^ Clinical studies have confirmed that silver needle can eliminate aseptic inflammation, improve blood circulation and relieve muscle spasm.^[11]^ The mechanism of silver needle therapy is similar to that of moxibustion, but moxibustion is heated by burning cotton balls and cannot control the temperature, while silver needles are heated by special equipment and the temperature can be set according to the patient's feedback.^[12]^ Silver needle therapy has achieved good results in low back pain, knee arthritis, ankylosing spondylitis and other diseases.^[12–14]^ However, there is a lack of clinical research on the efficacy and safety of silver needle therapy in the treatment of rheumatoid arthritis. Therefore, we intend to use this randomized controlled trial to evaluate the efficacy and safety of silver needle therapy in the treatment of rheumatoid arthritis.

## 2 Materials and methods

### 2.1 Study design

**Figure 1:**
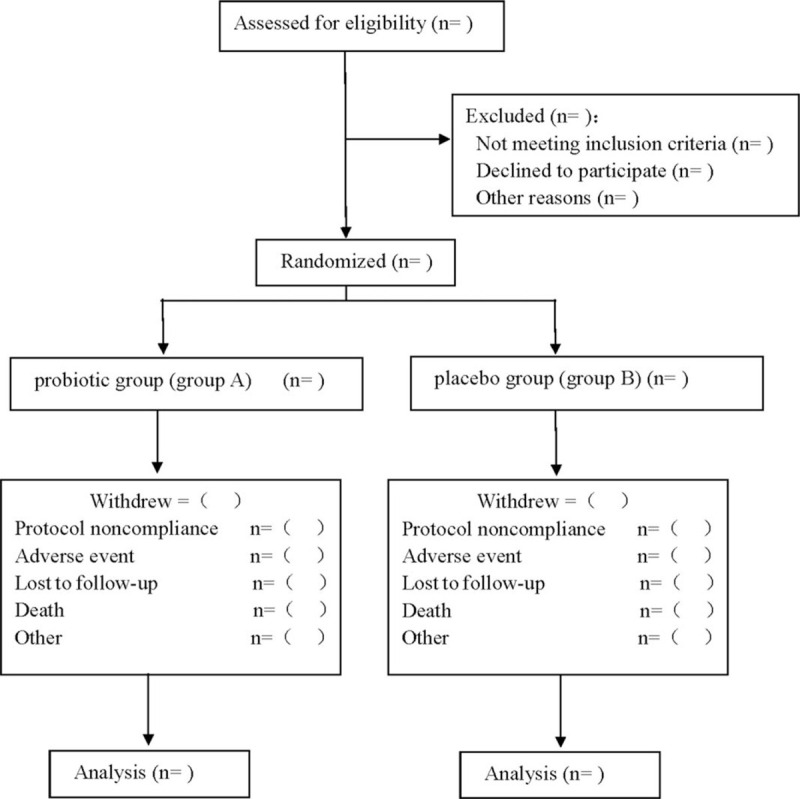
Flow diagram.

This is a prospective randomized controlled trial to study the clinical efficacy of silver needle therapy in the treatment of rheumatoid arthritis. This study will follow the comprehensive trial reporting standard.^[15]^ The flow chart is shown in Figure 1.

### 2.2 Ethics and registration

This research scheme is in line with the Helsinki Declaration and has been reviewed by the Clinical Research Ethics Committee of our hospital. This lab is registered at OSF (registration number: DOI 10.17605/OSF.IO/4X5QB). Before being randomly divided into groups, all patients are required to sign an informed consent form, and they can choose whether to continue the trial or discontinue at any time.

### 2.3 Sample size

The calculation of sample size is based on treatment, the mean value and standard deviation of TCM syndrome score. According to the results of the pilot study, the experimental group is 13.32 ± 4.30, and that of the control group is 15.83 ± 3.82. Set α = 0.025, one-sided test, β = 0.20. Calculated by PASS15.0 software, each group needs 43 participants, the estimated withdrawal rate is 20%, and each group will include 54 participants.

### 2.4 Patients

Inclusion criteria:

1. meet the diagnostic criteria of rheumatoid arthritis (refer to RA diagnostic criteria of ACR in 2010 and the EULAR^[16]^);
2. Age 18–70 years old, Rheumatoid Arthritis Disease Activity Score^[17]^ DAS-28 > 3.2, and in the active stage of the disease;
3. Patients who do not take related drugs or other treatment within one month;
4. Patients with strong compliance and signed informed consent.

Exclusion criteria:

1. Accompanied with important organ injury or malignant tumors, infectious diseases, blood system diseases;
2. Accompanied with other rheumatic diseases;
3. Pregnant and lactating women;
4. In the late stage of rheumatoid arthritis, severely deformed joints caused by disability, life cannot take care of themselves;
5. Allergic to the drugs used in this study.

### 2.5 Study design

Eligible participants are randomly assigned to either the treatment group or the control group in a 1:1 ratio using a central-network-based randomization tool. Random sequences are generated by SAS 9.3 software (SAS Institute, Cary, NC, USA) by independent statisticians who do not participate in the implementation of the experiment or statistical analysis. The clinical research coordinator enters participants’ information on the tablet and is given a random number. The research assistant gets the allocation of participants from the computer. Throughout the study, the research assistant is responsible for screening, recruiting participants, and assigning random numbers to participants who had been included. Given the operational nature of the intervention, participants and operators may be aware of random allocation. However, the evaluators of research results and the statisticians of data statistics and analysis do not know about the distribution.

### 2.6 Intervention measures

1. Treatment Group (silver needle treatment):
  a) Mark the tenderness point on the skin according to the pain site of the patient, which can be located on the muscle bundle, fascia, fascia or tendon. The needle point is determined by the central pain point, and the interval between each needle point is 1.0 cm – 2.0 cm. Finally, the needle point covers the whole painful area.
  b) After disinfection, 0.5% lidocaine is injected into the local anesthesia according to the needle point. Then insert the silver needle vertically or obliquely (the special silver needles which are 17 cm lengths of the needle body and 1 mm in diameter) into the needle point, usually through muscle or fascia attached to the bone surface or synovium. The patient will have soreness or swelling, indicating the correct position of the silver needle.
  c) Then the silver needle (ASTRONANTICS IN GOAL DIRECTION MED TECH Co., LTD., Shanxi, China) is connected to a special machine (YW-L1000; ASTRONANTICS IN GOAL DIRECTION MED TECH Co., LTD., Shanxi, China) and sets the heating temperature to ensure that the temperature of the silver needle entering the skin is about 42 °C for 25 min, often asking the patient how he feels to prevent scalds.
  d) After the needle is cooled, pull out the silver needle, disinfect the needle eye, wrap it with aseptic gauze, and do not touch water within 3 days. Once a week for 4 weeks.
2. the control group (conventional western medicine treatment): Celecoxib capsule (Pfizer Pharmaceutical Co., Ltd., STATE MEDICAL PERMITMENT No J20140072), 0.2 g / time, once a day; methotrexate tablets (Shanghai Xinyi Pharmaceutical Co., Ltd., STATE MEDICAL PERMITMENT No H3102064), 10 mg/, once a week; leflunomide tablets (Jiangsu Yabang Epson Pharmaceutical Co., Ltd., STATE MEDICAL PERMITMENT No H20080420) 20 mg/, once a day. The patients are treated continuously for 4 weeks.

### 2.7 Evaluation criteria and judgment of curative effect

1. TCM syndrome score: refer to the guiding principles of Clinical Research of New Drugs of Traditional Chinese Medicine (trial).^[18]^ The main symptoms and signs (including joint pain, joint swelling, limitation of joint activity, waist and knee soreness, fear of cold and cold, morning stiffness) are evaluated, and the scores are 0, 2, 4 and 6 respectively according to the severity, and the cumulative total score is the total score. The higher the total score, the more serious the illness.
2. The Health Assessment Questionnaire Disability Index (HAQDI).^[19]^ According to the difficulty of completing 8 life-related actions (including dressing and grooming, getting up, eating, walking, personal hygiene, touching, grasping, activities, etc.), they are divided into 4 grades, corresponding to 0, 1, 2 and 3 points respectively. The HAQDI value is calculated by accumulating the 8 scores and calculating the average value. The higher the score is, the worse the quality of life is.

1. DAS-28 score ^[17]^:
  a) ①DAS-28 score > 5.1 indicates high disease activity;
  b) ②3.2 < DAS-28 score ≤ 5.1 indicates moderate disease activity;
  c) ③2.6 < DAS-28 score ≤ 3.2 indicates low disease activity;
  d) ④DAS-28 score ≤ 2.6 indicates basic remission of the disease.
2. Laboratory examination: including serum calcium ion (Ca^2+^), ESR, hs-CRP, RF level.
3. Adverse reactions: discomfort related to treatment occurred during treatment.

The above observation indexes are collected on the day before and after treatment. All patients are followed up for 2 months, and the data are collected according to the same standard in the first and second month.

### 2.8 Data collection and management

The whole process of data collection and recording is carried out by one or two assistants. Personal information about potential participants and registered participants will be collected, shared and stored in a separate storeroom to protect pro-trial, in-trial and post-trial confidentiality. The access to the database will be restricted to the researchers in this study team.

### 2.9 Statistical analysis

The collected data are analyzed by SPSS 18.0 software. Chi-square test is used for counting data, mean ± standard deviation $\left(\bar{x}\pm \text{S}\right)$ is used for measurement data, independent sample *t* test is used for normal distribution, Mann–Whitney *U* test is used for skewed distribution, and the difference is considered to be statistically significant when *P* *<* .05.

## 3 Discussion

At present, the pathogenesis of rheumatoid arthritis is not fully understood, which may be related to autoimmune factors, infection and heredity.^[20]^ No matter the duration of the disease, early and effective treatment of RA patients can reduce their disability rate, control the disease activity of patients, help to prevent and delay the disease of patients, and is also the key to the treatment of rheumatoid arthritis.^[21,22]^ Clinical observation found that non-steroidal anti-inflammatory drugs, anti-rheumatic drugs and steroid drugs and other drugs in the use process, in addition to toxic side effects, but also showed a gradual failure trend, and some patients will develop drug resistance, seriously reducing the therapeutic effect.^[23]^ Therefore, it is urgent to explore safe and effective complementary and alternative therapies.

Silver needle is a therapeutic method evolved from traditional acupuncture, which combines the advantages of acupuncture and moxibustion. Clinical studies have shown that acupuncture combined with needle handle heating can significantly improve local blood circulation, reduce muscle spasm, relieve pain,^[24]^ and reduce the levels of interleukin-6 (IL-6), interleukin-8 (IL-8) and tumor necrosis factor-α (TNF-α).^[25]^ Animal studies have found that silver needle treatment can increase the content of β-endorphin for analgesia,^[26]^ but also significantly reduce the content of interleukin-6, play the role of analgesia and anti-inflammation.^[27]^ Since there is no standard clinical study to evaluate the efficacy of silver needle in the treatment of rheumatoid arthritis, we intend to evaluate its efficacy and safety through prospective randomized controlled trials. This work is beneficial to both patients and decision makers in clinical practice.

This study is a single-center study, and the regionalization of the population may have a certain impact on the results. Therefore, more multicenter, large-sample randomized controlled trials are needed to verify our conclusions.

## Author contributions

**Data curation:** Jinling Yu, Pingsheng Wang.

**Formal analysis:** Cui Nie, Bo Zheng.

**Funding acquisition:** Bo Zheng.

**Resources:** Pingsheng Wang, Cui Nie.

**Software:** Cui Nie, Bo Zheng.

**Supervision:** Cui Nie.

**Writing – original draft:** Jinling Yu, Pingsheng Wang.

**Writing – review & editing:** Jinling Yu, Bo Zheng.
